# Microtensile Bond Strength of Bioactive Pit and Fissure Sealants Bonded to Primary and Permanent Teeth

**DOI:** 10.3390/ma15041369

**Published:** 2022-02-12

**Authors:** Abdulelah AlQahtani, Yousef Al-Dlaigan, Ahmed Almahdy

**Affiliations:** Department of Pediatric Dentistry and Orthodontics, College of Dentistry, King Saud University, Riyadh 11545, Saudi Arabia; ydlaigan@ksu.edu.sa (Y.A.-D.); almahdy@ksu.edu.sa (A.A.)

**Keywords:** microtensile test, dental materials, dental sealants, bioactive materials, bond strength

## Abstract

Background: Sealing occlusal pits and fissures is an effective preventive measure against dental caries. Pit and fissure sealants (PFS) should be strongly bonded to the teeth to prevent partial or complete loss of the sealant, which may limit its preventive effect. Objective: The objective of the study was to compare the microtensile bond strength (μTBS) of bioactive resin-based sealants (Bio-RBS) and resin-based sealants (RBS), with and without the use of a bonding agent, to the enamel of primary and permanent teeth. Methods: One hundred and twenty caries-free primary molar specimens and 120 permanent molar specimens were divided to eight groups (30 specimens per group), both primary and permanent teeth were sealed with a Bio-RBS BioCoat^TM^ (Premier^®^, Plymouth Meeting, PA, USA) or with a RBS Clinpro^TM^ (3M ESPE, Saint Paul, MN, USA), with or without the use of a bonding agent (Prime & Bond NT; Dentsply, Inc., Charlotte, NC, USA). Half the specimens were aged with 5000 thermal cycles, and all specimens were tested for the μTBS and failure mode. Results: The mean μTBS of aged Bio-RBS was higher in permanent teeth than primary teeth, and the aging process reduced the μTBS of RBS more than that of Bio-RBS. Moreover, the addition of a bonding agent improved the μTBS of aged RBS in permanent teeth. Conclusion: We concluded that Bio-RBS exhibit superior μTBS than RBS when applied to permanent teeth.

## 1. Introduction

Dental caries is a multifactorial disease that occurs due to an altered balance between the demineralization and remineralization processes [1]. Although occlusal surfaces of the human teeth represent approximately 13% of the total tooth surface [2], the majority (80–90%) of dental caries are present in occlusal surfaces of permanent teeth in young patients [3]. Cleaning occlusal pits and fissures is problematic due to their plaque retentive nature. This makes them more prone to caries when compared to smooth tooth surfaces. Additionally, they are less protected by fluoride than other tooth surfaces [4].

During the early bell stage of tooth development, the occlusal fissure morphology of bicuspid and multicuspid teeth becomes mapped out by the internal enamel epithelium [5]. Pits are located at the intersection or ends of developmental grooves [6]. The morphology of the occlusal pits and fissures is not identical in all teeth [7]. The average depth of an individual fissure ranged from 120–1050 μm [8]. Four types of occlusal pits and fissures were identified by Nagano based on fissure morphology: V, U, I (Y1), and IK (Y2). V-shaped fissures are wider at the top than U-shaped fissures and gradually narrow toward the bottom. I-shaped fissures represent a very thin slit. Lastly, IK-shaped fissures resemble I-shaped fissures, but have a larger space at the bottom of the fissure [9].

Pits and fissures sealant (PFS) is one of the preventive tools that can be applied to prevent occlusal carious lesions, by creating a physical barrier preventing tooth impaction, plaque accumulation, and subsequent demineralization of the deep occlusal pits and fissures [10]. The application of bonding agents before applying PFS to increase its retention is a controversial issue. Current manufacturers’ instructions do not include using bonding agents. However, many studies have supported the use of a bonding agent before applying PFS. It was found that this technique improves the penetration of resin into occlusal fissures and enhances its retention [11]. This technique was first introduced to increase the retention of PFS when saliva contamination is expected, such as for sealing partially erupted teeth [12]. However, the possible increase of chairside time and cost led many to avoid using bonding agents before PFS application [13]. Moreover, some argued that this technique may reduce the PFS penetration into deep fissures as the physically weaker unfilled resin occupies the space between the sealant and enamel. However, when using a low-viscosity bonding agent with constant air drying, a delicate layer of the agent will remain, which will chemically bond to the PFS material [14].

Fluoride release is one of the advantages of glass ionomer-based PFS. However, they are inferior in physical properties compared to resin-based sealants (RBS). There is a paucity of data on the influence of monomers and fillers such as glass on remineralizing ion (fluoride, calcium, and phosphate) release from resin-based materials such as sealants [15]. Fluoride-containing RBS have higher retention rates and, thus, a better caries protection effect than GI-sealants [16]. However, another study found higher demineralization inhibition when glass ionomer-based PFS were used compared to both fluoridated and non-fluoridated RBS, no significant difference was found between fluoridated and non-fluoridated PFS, in terms of their effect on demineralization inhibition [17]. Another attempt to add remineralization ions to a RBS is by adding amorphous calcium phosphate (Aegis Opaque White, Bosworth Co., Ltd., Skokie, IL, USA), which exhibits its remineralization potential by supersaturation (an increase of calcium and phosphate ions within the carious lesion to levels that surpass those in oral fluids) and formation of apatite, especially in acidic environments [18]. A randomized clinical trial comparing the retention and caries prevention ability of amorphous calcium phosphate-containing PFS Aegis™ and moisture-tolerant fluoride-releasing PFS Embrace WetBond™ (Pulpdent co., Watertown, MA, USA) Aegis™ showed higher retention rates and better caries-prevention abilities [19].

In recent years, many attempts have been made to create a bioactive PFS material that is capable of preventing caries and exhibits the desired physical properties [20,21,22]. Recently, a new bioactive resin-based sealant (Bio-RBS) called BioCoat^TM^ (Premier^®^ Dental Products, PA, USA) has been introduced in the market. The company claims that the material releases ionic fluoride, calcium, and phosphate and that it has a similar retention to other RBS [23]. A recent study compared this material to other bioactive materials and found BioCoat^TM^ to have the highest flexural strength, elastic modulus, and remineralization abilities [24]. Another study found inhibition of enamel demineralization with experimental sealants containing bioactive glass in a cariogenic environment and concluded that PFS containing a bioglass filler is a promising dental material to prevent marginal or secondary caries at the marginal gap [25]. Another study assessed the rule of biosilicate enamel surface treatment on the bond strength of PFS and the effect of salivary fluids and found that it contributed to better bond strength, irrespective of substrate contamination [26]. A clinical trial investigated a bioactive self-etching sealant and found that it had a lower retention rate than a conventional RBS; however, both materials showed the same caries-prevention effect on newly erupted permanent molars [27].

In general, restorative materials are affected by intra-oral thermal changes that are induced by eating, drinking, and breathing. These thermal stresses induce mechanical stresses and increase gap dimensions. Thermal cycling procedures are used to mimic the intra-oral thermal changes [28]. The microtensile bond strength (μTBS) of RBS was found to be reduced after a 6-month aging period [29].

The aim of this study was to compare the μTBS of Bio-RBS and RBS applied to primary and permanent teeth, with and without the use of bonding agents. This study also studied the failure mode and the fissure morphology of all specimens. The null hypothesis was that there is no difference between the μTBS of Bio-RBS and RBS bonded to primary and permanent teeth, with and without the use of bonding agents, after aging by 5000 thermal cycles.

## 2. Materials and Methods

### 2.1. Sample

G*Power software (Version 3.1, University of Kiel, Kiel, Germany) was used to calculate the sample size. An effect size of 0.7 was assumed with 80% power. A total sample of 208 specimens (26 for each group) was required to test the difference at a 5% level of significance. However, we increased the sample to 30 Specimens for each group with a total sample of 240 specimens. Teeth were collected from patients attending the Pediatric Dentistry and Oral and Maxillofacial Clinics at Dental University Hospital, King Saud University, Riyadh, Saudi Arabia. All teeth extractions were part of their proposed treatment plans.

Sample collection was based on the following inclusion criteria: freshly extracted (1 month) primary and permanent molars from patients aged between 6 and 25 years old [30]; caries-free occlusal enamel. The exclusion criteria included: extracted teeth with developmental anomalies that affect the occlusal enamel surfaces, carious occlusal enamel surfaces, occlusal restorations, attrition, or erosion affecting the occlusal enamel surfaces, and teeth that had been stored for more than 1 month.

Upon receipt in the laboratory, adherent tissues were removed, and the teeth were cleaned and placed in fresh 0.5% chloramine-T and stored at 4–7 °C. All teeth were used within 1 month of extraction [30]. Samples were randomly allocated ensuring an equal number of specimens in each group using a web-based randomization software (Random.org).

### 2.2. Experimental Groups

The study included eight different groups. Moreover, each group was divided into immediately tested and aged specimens (Table 1).

### 2.3. Procedure

In every tooth, cusps were flattened leaving the fissures intact. After that, the specimens were sealed in room conditions (23 ± 2 °C) as follows: First, the specimens were cleaned using a manual medium toothbrush followed by acid-etching of the surface with 35% phosphoric acid (Ultra-Etch^TM^; Ultradent Products, Inc., South Jordan, UT, USA) for 20 s, followed by rinsing for 5 s with water. Then, the specimens were air-dried for 10 s; care was taken to avoid desiccation of the enamel surface.

In the bonding groups (2, 4, 6, and 8) bonding was achieved using a two-step etch-and-rinse bonding system (Prime & Bond NT; Dentsply International, Inc., Charlotte, NC, USA) and was activated using an Elipar S10 LED curing light (3M ESPE, St. Paul, MN, USA) with a wavelength of 430 nm for 20 s, according to the manufacturer’s instructions. Bio-RBS material (BioCoat^TM^ Premier^®^ Dental Products, Plymouth Meeting, PA, USA) was applied in groups 5, 6, 7, and 8, while in groups 1, 2, 3, and 4, RBS material (Clinpro^TM^ 3M ESPE, St. Paul, MN, USA) was applied in 5 mm thickness. Polymerization was achieved with an Elipar S10 LED curing light for 20 s. After that, the surface was sectioned using a slow-speed water-cooled diamond blade (MetLab Technologies Limited, New York, NY, USA) to produce 1 mm^2^ specimens.

Half of the specimens were tested immediately, while the other half were aged with 5000 thermal cycles between 5 °C and 55 °C with a dwell time of 30 s, using a Thermocycler 1100It (SD Mechatronik, Feldkirchen-Westerham, Germany), which is equivalent to a 6-months aging period. After that, the specimens were bonded to a custom-made jig and tested using a microtensile device MTD-500 (SD Mechatronik, Feldkirchen-Westerham, Germany) at a speed rate of 0.5 mm/min. The force needed to break the bond was recorded in megapascals (MPa). The materials used in this study and their composition are explained in Table 2.

**Figure 1:**
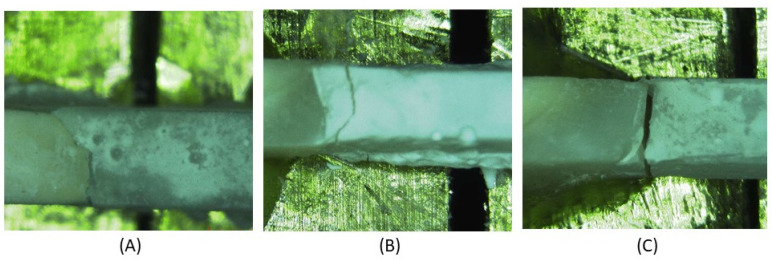
Examples of the different failure modes (**A**) adhesive, (**B**) cohesive, (**C**) Mixed.

For pre-test failures, a score equal to the mean between 0 MPa and the lowest measured value in the same experimental group was recorded. For manipulation errors, occurrence and number were noted, and teeth were excluded from the dataset. Each specimen was examined using a Stereo 80 Widefield Microscope (SWIFT Instruments, Inc., Boston, MA, USA), to evaluate the failure mode, which was decided by the residual resin on the enamel surface to determine the bond failure. The failure modes were classified as ‘adhesive failure mode’, ‘cohesive failure mode’, or ‘mixed failure mode’. When a break existed at the interface between the enamel and the resin, it was classified as an adhesive failure. If the failure existed predominantly within the resin, it was classified as a cohesive failure. Mixed failure was determined when part of the resin remained attached to the enamel (Figure 1). The same microscope was used to evaluate the fissure type following Nagano’s classification [9]. The fissures were classified as V, U, I, and IK. For analysis, fissures V and U were combined in a group as ‘shallow fissures’, while I and IK fissures were combined as ‘deep fissures’.

### 2.4. Statistical Analysis

Data were analyzed using SPSS statistical software (Version 25.0, IBM Inc., Chicago, IL, USA). One-way ANOVA and Tukey post hoc tests were used for multiple comparison of μTBS between the different groups, while an independent t-test was used to compare the μTBS between the immediate and aged specimens in each group. For the failure mode, a Pearson chi-square test was used. Point-biserial correlation was used to study the effect of fissure type on μTBS. The significance level was set at α = 0.05.

## 3. Results

**Figure 2:**
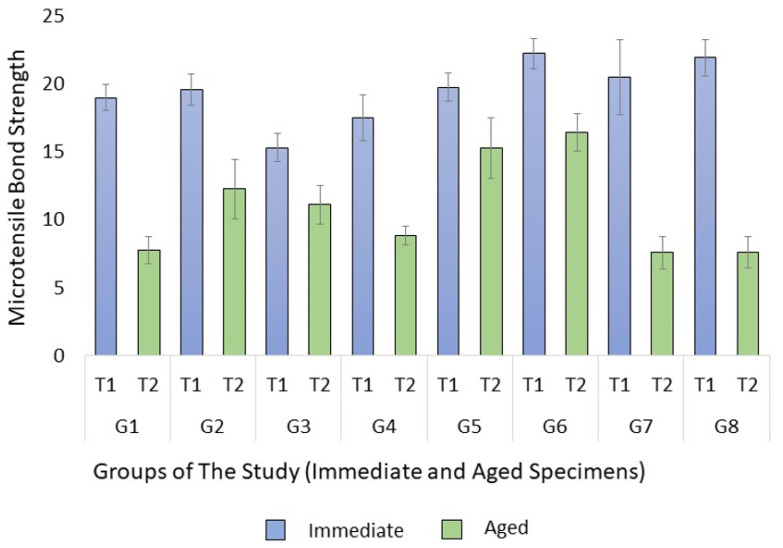
Means of microtensile bond strength (μTBS) in MPa with standard error of the mean.

The analysis of variance test showed a significant difference between the groups in both immediate (*p* = 0.028) and aged (*p* < 0.001) specimens. When the specimens were tested immediately, the multiple comparison test revealed that primary teeth sealed with a non-bonded RBS (Group 3) had a significantly lower μTBS than both permanent and primary teeth sealed with bonded Bio-RBS (Groups 6 and 8) (*p* = 0.012 and 0.042, respectively). No significant difference was found between other groups. However, when tested after the thermocycling aging process, the test showed a significantly higher μTBS for the permanent teeth sealed with non-bonded Bio-RBS (Group 5) than with regular RBS (Group 1) (*p* = 0.01). It was also significantly higher compared to primary teeth sealed with non-bonded Bio-RBS (Group 7) and bonded Bio-RBS (Group 8) (both *p* = 0.009 and 0.009). Moreover, there was a borderline significance, as the μTBS of permanent teeth sealed with non-bonded Bio-RBS (Group 5) was higher than primary teeth sealed with bonded RBS (*p* = 0.054). The μTBS of permanent teeth sealed with bonded Bio-RBS (Group 6) was significantly higher than permanent teeth sealed with non-bonded RBS (Group 1) (*p* < 0.001). It was also significantly higher than primary teeth sealed with bonded RBS (Group 4) and bonded Bio-RBS (Group 8) (*p* = 0.01 and 0.001, respectively). The means of μTBS in MPa are shown in Figure 2. Moreover, the independent *t*-test showed a significantly higher μTBS in immediate specimens than aged specimens within all groups, except group 5 (*p* = 0.08).

**Figure 3:**
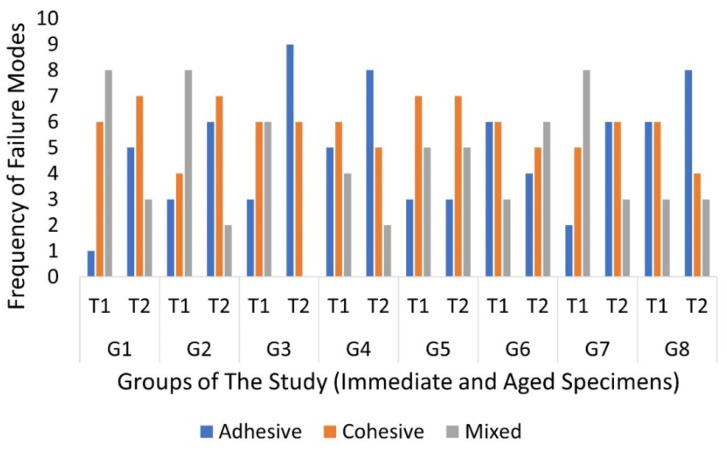
Frequency of the different failure modes in both times of testing.

For the failure mode, there was no significant difference when comparing between the immediate specimens (*p* = 0.483). In addition, there was no significant difference within the aged specimens (*p* = 0.409). When tested immediately, adhesive failure occurred in 20.2% of the total specimens, cohesive failure occurred in 38.3%, and the percentage of mixed failure was 37.5%. In the aged specimens, adhesive failure occurred in 40.8%, cohesive failure occurred in 39.2%, and the percentage of mixed failure was 20%. The occurrence of the different modes in each group is shown in Figure 3. Within primary teeth sealed with non-bonded RBS (Group 3), there was a significant difference between the immediate and aged specimens (*p* = 0.01); however, there was not any significant differences in terms of the time of testing within any of the other groups. Pre-test failures and manipulation errors are shown in Table 3. There was no significant difference between the groups in terms of fissure type (*p* = 0.26), and the point-biserial correlation found no effect of the different fissure types on the μTBS.

## 4. Discussion

The use of PFS for the prevention of occlusal caries is advocated by many dental associations, including the American Dental Association (ADA) and The American Academy of Pediatric Dentistry (AAPD), in their evidence-based practice guidelines [10,31]. However, the frequent partial or complete loss of PFS has led many dentists to advocate other prevention techniques, such as topical application of fluoride. In their clinical trial, Chestnutt et al. found caries development after 3 years of follow-up in 17.5% of teeth in the fluoride varnish group, compared to 19.6% of teeth in the PFS group, which was not statistically significant [32]. However, teeth with full or partial loss of PFS are not at a higher risk of developing dental caries than teeth that were never sealed. In fact, they may be more resistant to caries than non-sealed teeth, and the benefits of the application of PFS exceed the potential risks, even if follow-up appointments could not be ensured [33].

After immediate testing, it was found that primary teeth sealed with non-bonded RBS had the lowest μTBS. Thermal cycling provides an accurate simulation of the effect of the aging process [28]. When tested after the aging process, the means of μTBS of all groups had decreased. This shows the effect of frequent thermal changes on the bond between the PFS material and enamel. It was also found that the mean μTBS of Bio-RBS was higher than the mean μTBS of regular RBS, only in the aged specimens. This means that the Bio-RBS performed better than RBS in permanent teeth without the use of bonding agents.

In general, the μTBS of sealants applied on permanent teeth was higher than primary teeth. Many studies reported lower bond strength of PFS when applied on primary teeth compared to permanent teeth [34]. This could be due to the presence of a prism-less superficial layer on the enamel of primary teeth that prevents penetration of the PFS material, affecting its bond to enamel [35].

Cohesive failures were almost the same at both times of testing. Moreover, the immediate specimens showed more mixed failures, while the aged specimen showed more adhesive failures, this clearly showed the effect of the aging process on the bond at the resin–enamel interface. This could be explained by the frequent change of temperature, which causes mechanical stresses that widen the gaps within the resin–enamel interface by pumping fluids in and out of that gap [28].

Occlusal morphology is an important factor when deciding if a tooth needs to be sealed; deep occlusal pits and fissures are more prone to plaque accumulation and subsequent decay [14]. A study evaluated 160 sound human permanent molars and found that fissure type Y1 was found the most (33.12%), followed by V (26.88%), and U and Y2 types (20% each). For further analysis, they combined fissure types U and V in a group of shallow fissures, and fissure types Y1 and Y2 were combined in a group of deep fissures. Regarding the prevalence of different fissure types, there was no statistically significant difference among the different groups [36]. In our study, we found that the depth of the occlusal fissure was not an influential factor on the μTBS. As discussed previously, the occlusal morphology’s main influence is on the penetration of the resin into the enamel [11].

A recent study investigated the flexural strength, elastic modulus, and remineralization abilities after 7 days of pH-cycling of bioactive and non-bioactive sealant materials and found that BioCoat^TM^ had the highest amount of fluoride, calcium, and phosphate ions, and the tinniest demineralization area; moreover, they were also superior in terms of their flexural strength and modulus of elasticity [24]. This supports choosing BioCoat^TM^ as the material representing Bio-RBS in this study. The findings of the current study show that the μTBS of Bio-RBS in the aged specimens was better than RBS. This could be explained by the presence of calcium and phosphate ions in both the enamel structure and the Bio-RBS material. The application of a bonding agent before Bio-RBS may create a physical barrier, preventing a chemical bond between Bio-RBS and enamel. The concept of Bio-RBS may pave the way for a material that has the ability to prevent demineralization and that has the physical properties to withstand the oral environment and masticatory forces.

Moisture control is an important factor that increases the success of PFS. Isolation techniques vary widely with cotton roll isolation being the most common among pediatric dentists [37]. A study evaluated three PFS application techniques on different enamel conditions: uncontaminated, saliva-contaminated, and water-contaminated. It was found that under salivary contamination, the addition of a water-based self-etching adhesive under PFS gave better shear bond strength than ethanol-based self-etching adhesive and etching with phosphoric acid, without adding a bonding agent [38]. In the current study, the etch-and-rinse technique was used before adding a bonding agent, which has been shown to give better bonding results than self-etch bonding systems [39,40,41,42].

This study provided baseline information for the μTBS of Bio-RBS bonded to both primary and permanent teeth. Moreover, it studied the effect of fissure morphology on the μTBS. It also compared aged specimens with immediately tested specimens. Nevertheless, there is a need for further studies to compare the Bio-RBS to other available PFS materials, not only in their μTBS but also in their micro-shear bond strength, microleakage, and anti-bacterial effects on the composition of occlusal enamel. The effect of the etching time and the type of bonding is another factor that could be investigated using this new material. Moreover, there is a need for clinical studies to determine the success/retention rate of this material with long-term follow-ups. This study will be followed by a study investigating the interface morphology of the Bio-RBS applied to primary and permanent teeth.

## 5. Conclusions

The following can be concluded from this study:

- The μTBS of aged Bio-RBS was higher in permanent teeth than primary teeth.
- The inimical effect of the aging process on the μTBS of RBS is higher than its effect on Bio-RBS.
- The addition of a bonding agent improved the μTBS of aged RBS in permanent teeth.

## Figures and Tables

**Table 1:** The experimental groups of the study.

Group	Tooth Type	Sealant Material	Bonding
1	Permanent Molars	Clinpro™	Non-bonded
2	Permanent Molars	Clinpro™	Bonded
3	Primary Molars	Clinpro™	Non-bonded
4	Primary Molars	Clinpro™	Bonded
5	Permanent Molars	BioCoat™	Non-bonded
6	Permanent Molars	BioCoat™	Bonded
7	Primary Molars	BioCoat™	Non-bonded
8	Primary Molars	BioCoat™	Bonded

**Table 2:** The materials used in this study, their manufacturer, and composition.

Material	Manufacturer	Composition
Acid Etch	Ultra-Etch^TM^ etchant; Ultradent Products, Inc., South Jordan, UT, USA	35% Phosphoric acid
		Highly dispersed Silicon
		Dioxide
		Colorant Water
Bonding Agent	Prime & Bond^®^ NT; Dentsply International, Inc., Charlotte, NC, USA	Urethane dimethacrylate (UDMA)
		Trimethacrylate
		Phosphoric acid modified acrylate resin (PENTA)
		Highly dispersed silicon dioxide
		Camphorquinone (photoinitiator)
		Ethyl-4(dimethylamino)benzoate
		Butylated hydroxy toluene (BHT)
		Cetylamine hydrofluoride
		Acetone
Pits and Fissures Sealant	Clinpro^TM^ 3M ESPE, St. Paul, MN, USA	Bis-GMA
		Triethylene glycol dimethacrylate,
		Photo-Initiator based on camphorquinone
		Tertiary amine
		Iodonium salt
		Silane-treated fumed silica Titanium dioxide (opaquer)
		Patented organic fluoride salt
		Rose Bengal pink dye
	BioCoat^TM^ Premier^®^ Dental Products, Plymouth Meeting, PA, USA	Bis-GMA
		Barrium Aluminoborosilicate
		Triethylene glycol dimethacrylate
		Calcium Donor
		Phosphate Donor
		Fumed Silica
		Photo-Initiator

**Table 3:** Frequency of manipulation errors and percentage of pre-test failures in each group.

Group	Immediate Specimens (T1)		Aged Specimens (T2)	
	Manipulation Errors	Pre-Test Failures	Manipulation Errors	Pre-Test Failures
1	0	6.7%	0	20%
2	0	6.7%	0	20%
3	1	0%	0	0%
4	2	0%	1	6.7%
5	1	6.7%	1	13.3%
6	0	0%	0	0%
7	0	0%	0	6.7%
8	0	0%	0	6.7%

## Data Availability

Data are available upon request.
